# Objective Assessment of Binaural Benefit from Acoustical Treatment in Real Primary School Classrooms

**DOI:** 10.3390/ijerph20105848

**Published:** 2023-05-17

**Authors:** Greta Minelli, Giuseppina Emma Puglisi, Arianna Astolfi, Christopher Hauth, Anna Warzybok

**Affiliations:** 1 Department of Energy, Politecnico di Torino, 10129 Torino, Italy; 2 Medizinische Physik and Cluster of Excellence Hearing4All, Carl von Ossietzky University of Oldenburg, D-26111 Oldenburg, Germany

**Keywords:** speech intelligibility, binaural listening, classroom acoustics, reverberation time, noise

## Abstract

Providing students with an adequate acoustic environment is crucial for ensuring speech intelligibility in primary school classrooms. Two main approaches to control acoustics in educational facilities consist of reducing background noise and late reverberation. Prediction models for speech intelligibility have been developed and implemented to evaluate the effects of these approaches. In this study, two versions of the Binaural Speech Intelligibility Model (BSIM) were used to predict speech intelligibility in realistic spatial configurations of speakers and listeners, considering binaural aspects. Both versions shared the same binaural processing and speech intelligibility backend processes but differed in the pre-processing of the speech signal. An Italian primary school classroom was characterized in terms of acoustics before (reverberation, T20 = 1.6 ± 0.1 s) and after (T20 = 0.6 ± 0.1 s) an acoustical treatment to compare BSIM predictions to well-established room acoustic measures. With shorter reverberation time, speech clarity and definition improved, as well as speech recognition thresholds (SRTs) (by up to ~6 dB), particularly when the noise source was close to the receiver and an energetic masker was present. Conversely, longer reverberation times resulted (i) in poorer SRTs (by ~11 dB on average) and (ii) in an almost non-existent spatial release from masking at an angle (SRM).

## 1. Introduction

Children’s learning abilities are influenced by the acoustic quality of the environments where they spend most of the time during their everyday life. This is particularly true at the first stages of education [1,2,3,4,5,6,7], when children need classrooms with good speech intelligibility for tuning out competing sounds and tuning into speech. Previous studies evidenced that speech intelligibility, which is defined as the percentage of correctly heard speech items with respect to the overall [8], is reduced through the presence of competitive effects of reverberation and background noise [2,9]; the higher the reverberation and noise, the lower the speech intelligibility. These factors not only impair speech intelligibility but also have a detrimental effect on academic performance [10,11,12]. Additionally, good classroom acoustics increase the degree of satisfaction of the acoustical quality [13], lowers the perception of noise disturbance and improves the perception of well-being at school [14]. In order to guarantee good classroom acoustics, thresholds of acoustical parameters have been investigated [15] and new benchmark values have been included in the most recent standards on classroom acoustics at national and international levels [16,17,18,19,20]. However, they essentially refer to a monaural listening perspective and, thus, cannot predict potential advantages produced via binaural listening processes.

Besides ambient noise and reverberation, speech intelligibility depends on the spatial configuration of the acoustic sources, i.e., the mutual position between speech-source, noise-source and listener, and it can benefit from binaural listening thanks to spatial hearing. In fact, the cocktail party effect [21] describes how listeners can focus on a specific speech signal even in challenging sound environments, such as classrooms in which speech intelligibility is corrupted by reverberation and ambient noise, thanks to the spatial separation of the target and the masker. The ability to use the difference in spatial position between a target and a masker to discriminate the target is referred to as spatial release from masking (SRM) [22]. SRM has been attributed to both binaural processing and head-shadow effect [21,22,23,24,25], and can result in an improvement of up to 12 dB in speech recognition threshold (SRT), i.e., the signal-to-noise ratio (SNR) required to achieve a 50% correct recognition score. In practice, SRM is calculated as the difference between an SRT measured under co-located target and noise source positions and an SRT measured under spatially separated target and noise positions. Indeed, several studies already proved that such improvement can occur when a noise source is spatially separated at an angle with respect to a listener’s ears and with respect to the target source, too [22,26,27]. However, the benefits from binaural cues that are gained under advantageous spatial configurations were proved to be reduced in reverberant conditions [28,29]. With respect to the variation of speech intelligibility due to target-to-receiver distance, worsening of SRTs at increasing distances was found for different acoustic conditions [30]; however, questions are still open. Puglisi et al. [30] performed investigations in primary school classrooms with a reverberation time at mid-frequency of 0.4 s and 3.0 s, where the acoustic field was approximated as semi-reverberant. They expected worsening SRTs as a decrease in level by 3 dB per double distance (dB/dd), but instead, they obtained this decrease to be ~2 dB/dd. This outcome was obtained regardless of the acoustic condition and noise type (i.e., either informational with semantic content, or energetic, which is speech-shaped in its frequency distribution but does not have semantic content). This result was also corroborated in Astolfi et al. [31], where the decrease per double distance of the speech level in classrooms characterized by different acoustic conditions was found to be ~−2 dB/dd.

To date, the majority of research has investigated how noise and reverberation influence speech intelligibility and spatial release from masking in controlled laboratory settings [32,33,34,35]. However, only a limited number of investigations have examined these factors in real environments, e.g., where noise and reverberation are ecologically valid [30,36]. Binaural speech intelligibility has been also investigated through prediction models [26,29,37,38,39,40,41]. Many of the binaural speech intelligibility models showed high correlations between predicted and measured SRT for different spatial configurations of noise and speech sources in anechoic and reverberant rooms and resulted in a relatively small mean absolute prediction error of 3 dB, considering normal hearing subjects [29]. In this study, the binaural speech intelligibility model (BSIM) [29] was used. Since the BSIM cannot satisfactorily predict the decrease in speech intelligibility with increasing distance resulting from the detrimental effect of late reflections on the speech signal, Rennies et al. [28] introduced and evaluated three extensions which considered this aspect. Here, the approach employed involved dividing the speech signal into a beneficial component and a detrimental one, where the detrimental part of the speech signal was added to the noise. In this way, the detrimental part of the speech signal effectively decreased the SNR at the input of the BSIM. 

A model capable of evaluating the variation of intelligibility in real acoustic conditions can bring great advances to the design of classroom acoustics. As an example, it allows to assess the benefit of acoustical treatment and to organize the classroom to suit specific priorities. Different configurations combining absorptive and diffusive surfaces could be examined, quickly and effectively orienting the achievement of optimal acoustics through using an efficient design in terms of correct location, quantity, and geometry of acoustic surfaces.

Within this study, the BSIM is evaluated as an objective measure of speech intelligibility in classrooms and as an objective indicator of the effectiveness of acoustical treatment in terms of binaural speech intelligibility. Two primary school classrooms, identical in size but significantly different in terms of acoustics, were selected. On the one hand, one classroom was subjected to acoustical treatment and considered as an example of a learning environment with good acoustics because of short reverberation time and high speech clarity. On the other hand, the second classroom was not acoustically treated and was characterized by poor acoustics. To evaluate speech intelligibility, binaural room impulse responses (BRIRs) were measured at various distances from a target source that was fixed at a typical teacher’s position. Additionally, a source that produces masking noise was positioned both in spatially co-located and in spatially separated positions from the listener’s ears. Using this experimental setup that incorporates spatial considerations, the objective was to determine to what extent the location of the masking noise, in terms of both distance and angle, impacts the receiver’s speech intelligibility. The speech and noise anechoic stimuli were convolved with the BRIRs, and the resulting output was fed into the BSIM [28,29] to estimate speech intelligibility in terms of SRTs. Based on the predicted SRTs, speech intelligibility and SRM were compared between the two classrooms. Additionally, reverberation time (T20), early decay time (EDT), speech clarity (C50), and speech definition (D50) were derived from the measured monaural room impulse responses, and then were put in relation to the predicted SRTs.

## 2. Materials and Methods

### 2.1. Classrooms

Two classrooms in the same school building were subject to measurement campaigns to assess their acoustics from both monoaural and binaural perspectives. Although the architectural characteristics, including geometry and finishes, are the same for both classrooms, their acoustical properties differ because one of them had undergone an acoustical treatment. For the sake of clarity, due to the similarity of the two classrooms, they will be treated as a single one. In each of them, the ante operam (AO) and post operam (PO) conditions were considered when measurements and predictions were performed before and after the acoustical treatment, respectively. The classrooms have a rectangular shape, with a plan of 6.7 m × 8.4 m, and an overall volume of 258 m^3^. The classrooms have three windows that face a courtyard, which is adjacent to a road with moderate traffic. The floor is primarily covered with Venetian tiles, while the walls and ceiling are plastered in the case of the AO condition. During the measurement sessions, the classrooms were furnished with desks and chairs, bookshelves along the lateral walls, and blackboards. In the PO classroom, glass-fiber absorbent panels (absorption coefficient averaged at 0.5–1 kHz, α_0.5–1kHz_ = 1) were added to the left longitudinal (9.7 m^2^) and rear (16.2 m^2^) walls, and a glass-fiber countertop (α_0.5–1kHz_ = 0.95) was installed on the ceiling (56.3 m^2^). Measurements were performed outside of school hours, and to simulate the presence of 23 children seated at their desks, 100% polyester fiber panels (0.6 m × 0.6 m × 0.05 m each) were employed. This method to simulate occupancy in the rooms was adopted from past studies [4,42]. In particular, the absorptive panels made of polyester fiber were dimensioned in order to replicate the equivalent absorption area of seated children, that is, of about 0.35 m^2^ at 1 kHz. 

### 2.2. Acoustic Measurements 

#### 2.2.1. Monoaural Measurements

The purpose of the monoaural measurements was to characterize the room’s acoustics in accordance with EN ISO 3382-2:2008 [43]. Measurements were performed using a calibrated sound level meter (NTi XL2 Audio, Schaan, Liechtenstein) and a directional source (NTi TalkBox Audio, Schaan, Liechtenstein). Four receiver positions along the central axis were recorded, which were located at 1 m, 2.2 m, 3.6 m, and 6.2 m from the directional source, always at a height of 1.2 m from the floor. The directional source remained in the same position, placed on the classroom’s central axis, 1 m from the frontal wall and 1.5 m from the floor. The sound level meter recorded the exponential sine sweep signals emitted by the TalkBox, from which room impulse responses were then deconvolved.

The Italian UNI 11532-2:2020 standard [20] was considered as the most recent reference for reverberation time and speech clarity to assess the suitability of the acoustical treatment to the aim of making the classrooms compliant with the requirements for learning environments. This standard is voluntary for private constructions, but mandatory in the public sphere. Regarding the reverberation time parameter, it allows for evaluations in different frequency bands, while in the case of speech clarity it allows for averaging them and, thus, characterizing the room with one value. The latter should make comparisons across the rooms/cases easier. So, to the aim of the present study, reverberation time and speech clarity values are both reported only as overall values in order to provide readers (either academics, researchers, or professionals) with synthetic values that can be easily compared with those from other studies or cases. In particular, reverberation time (T20, s) was considered as optimal for the involved classroom if it was approximately 0.5 s [20], early decay time (EDT, s) was optimal if comprehended in the range of 0.3 s to 0.7 s [44], and speech clarity (C50, dB) and speech definition (D50, dB) were optimal if greater than 2 dB [20] and in a range of 0.86 to 1.0 [45], respectively. As far as frequency averaging is concerned, T20 was averaged between 125 Hz and 4 kHz, C50 was averaged between 500 Hz and 2 kHz, while EDT and D50 were averaged between 500 Hz and 1 kHz.

#### 2.2.2. Binaural Measurements

**Figure 1:**
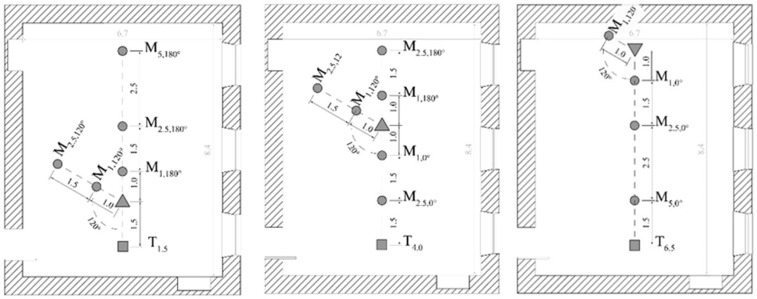
Schemes of the spatial configurations. Squares represent the target source (T), triangles identify the receiver, and circles indicate the masker positions (M). Specification of linear and angular distance from the receiver are indicated in subscript for target and maskers: for example, T_1.5_ indicates that the target is 1.5 m away from the receiver, while M_5, 180°_ indicates that the position of the masker is 5 m away from the receiver and with an angle of 180°.

The objective characterization of classrooms for the identification of challenging listening scenarios was carried out through performing binaural measurements in both AO and PO conditions. The target source, the noise source, and the receiver were positioned as shown in Figure 1. In particular:

- The target source (T) consisted of a NTi Audio TalkBox, which exhibits the speech directivity pattern of a human voice. The T source was placed 1 m from the rear wall on the central axis of the classroom, at a height of 1.5 m from the floor.
- The masking source (M) consisted of a Larson Davis omnidirectional (dodecahedral) sound source. It was set at 1.5 m height at several positions that varied in azimuth with respect to the receiver’s ears. In particular, M was placed in co-located positions (i.e., at 0° and 180°) and in a separated position (i.e., at 120°), and then, where possible due to spatial availability, at increasing distances (i.e., at 1 m, 1.5 m and 2.5 m).
- The receiver (R) consisted of a Brüel & Kjær (B&K) Head and Torso Simulator (HaTS), which allowed for the recording of binaural room impulse responses (BRIRs). The HaTS was placed in the classroom in order to guarantee that its ears were at 1.5 m from the floor. Furthermore, it was then placed at increasing distances from the T source, i.e., at 1.5 m, 4.0 m, and 6.5 m, referred in the following as T1.5, T4.0, and T6.5, respectively.

T, M, and R were positioned in the classroom according to the most complex configurations already explored in the available literature with regard to the effect of acoustics and spatial distribution of sources on SRT and SRM. 

In Figure 1, the left-hand configuration shows the close target-to-receiver distance (T1.5), which corresponds to the first row of students’ desks and, thus, to the most advantaged condition under evaluation. Here, M is placed separated and co-located with respect to R, i.e., at 120° and 180° of the azimuth, respectively, and at increasing distances. This allows for the comparison with the outcomes of Westermann and Buchholz [36], who studied the effect of noise distance on an axis (from 1 m to 10 m). As far as the influence of spatial configurations and acoustics on SRM are concerned, it is expected that SRM values will reduce in AO and, therefore, under longer reverberation time and at increasing distances between M and R.

The central scheme shown in Figure 1 corresponds to the central row of desks with a target-to-receiver distance of 4 m (T4.0) and is assumed to be the most disadvantageous listening condition. Again, M is placed at increasing distances and at co-located and separated azimuths. In such a spatial configuration, it is possible to assess speech intelligibility variations while also evaluating the effect of the M source when in front or behind R ears, since changes may introduce significant differences due to the spectral cues [22].

Last, the left-hand configuration in Figure 1 shows the very far target–receiver condition (T6.5). This distance between T and R approximately corresponds to the last row of student desks and it is assumed that, although the distance itself is the largest, speech intelligibility can be improved compared to T4.0 due to the reflections of the rear wall. In line with the conditions previously described, M is placed at increasing distances and at different azimuths. Particularly in this case, the co-located M condition corresponds to a situation where the source is placed in between T and R, i.e., at 0°.

### 2.3. Binaural Speech Intelligibility Model (BSIM) 

#### 2.3.1. Model Description

The BSIM [29] was used to predict SRTs for different combinations of noise source and receiver positions in both the untreated and acoustically treated room (AO and PO, respectively).

The BSIM requires separate clean speech and noise signals for the left and right ear, which were generated through convolving them with the corresponding BRIRs. A stationary, speech-shaped noise of the Italian matrix sentence test [46] was used. In the model, the signals are divided into separate frequency bands using a gammatone filterbank [47] ranging from 146 Hz to 8300 Hz in 30 ERB spaced frequency bands simulating the frequency selectivity of the human auditory system. After that, the equalization-cancellation (EC) [48] mechanism is used as a model of human binaural processing. In the EC mechanism, the interaural differences in level and time, namely the ILD and ITD, are equalized in each frequency band independently. In the cancellation step, the equalized left ear channels are subtracted from the equalized right ear channels. The equalization of ITDs and ILDs is optimized such that the SNR is maximized after applying the cancellation step. The accuracy of the equalization is limited by internal noise, which mimics the limited abilities of the human auditory system in binaural cues processing in the time (delay error) and level (gain error) domains. The gain error controls the overall balance between time- and level-dependent terms. The delay error has an influence on binaural processing at high frequencies and simulates the decreasing phase coherence of the auditory nerve towards high frequencies. The variances of the binaural processing errors in the model were adapted from vom Hövel [38], who derived them from simulations of pure tone binaural masking level differences. If the target and noise signals differ in their ITDs and ILDs, which is the case if they are spatially separated, the EC mechanism can substantially improve the SNR, especially at low frequencies. In the next step, the EC-maximized SNRs are compared to the monaural SNRs of the left and the right ear in each frequency band and the best SNR of all three alternatives is chosen. In the last step, the speech intelligibility index (SII) [18] performs a weighting of the band-specific SNRs to mirror human speech perception, which are then integrated over frequency and transformed to an index value between 0 and 1. The resulting SII values can then be mapped to SRTs.

#### 2.3.2. Model Calibration

In order to map the SII values to an SRT, a reference condition needs to be defined to calibrate the BSIM. This procedure requires an empirical SRT, which is usually measured in anechoic conditions with co-located target and masker sources placed in front of the listener at the same distance. Here, a different approach was used, because the acoustical sources for speech (TalkBox) and noise (dodecahedron) were not the same and no SRTs obtained with human listeners were available. Instead, the BSIM was calibrated to the PO condition, where speech arrives from the target source position and the noise is located 1 m behind the listener (M3). This situation is most similar to the anechoic situation typically used for calibration since the binaural effects are almost negligible and the target is not influenced by reverberation due to a very short target–receiver distance. The calibration obtained in this situation was kept constant for all measurement conditions.

The reference SII, corresponding to the SRT for 50% speech recognition, was set to 0.22, which corresponds to empirical SRT50 in anechoic and co-located speech/noise conditions for the Oldenburg sentences test in noise [29,49,50]. It is important to note that the relative differences across conditions which are of main interest here are not affected by choosing the reference SII.

#### 2.3.3. Comparison between the BSIM Models of Beutelmann et al. (2010) [29] and Rennies et al. (2011) [28]

The main difference between the model versions described in Beutelmann et al. [29] and Rennies et al. [28] is the pre-processing of the target speech. In [29], the target speech is convolved with the corresponding BRIR in order to produce the binaural input to the model. This model is well-suited for speech intelligibility simulations as long as the influence of room acoustics on noise dominates the results. However, the model is not able to simulate the detrimental effect of late reflection on the speech signal itself. This limitation is overcome in the approach of Rennies et al. [28], in which the target BRIR is first divided into an early part (≤100 ms), and a late part (>100 ms). Then, the speech signal is convolved with both parts of the BRIR, where the speech convolved with the early part is considered the target signal, and the speech convolved with the late part is added to the noise. In this way, the amount of late reverberation present in the speech signal is treated as detrimental and affects the SNR at the input, where more energy in the late part leads to more energy in the noise signal. Effectively, it decreases the SNR and, through that, leads to a higher predicted SRT. In spatially separated conditions, the effect is even more complex, since in addition to the changes in the SNR, the late reflections affect also the correlation of the noise signals presented to both ears. This can lead to less effective EC processing and, thus, to increased SRT and/or reduced SRM.

## 3. Results and Discussion

### 3.1. Classroom Acoustics Results

Table 1 displays the acoustic parameter values measured for both the conditions before and after the acoustical treatment, i.e., AO and PO, respectively. In the PO condition, all the investigated parameters conform to the standards. Reverberation time (T20_0.125–4kHz_) falls within the range of values recommended as the optimum for teaching and communication rooms by UNI 11532-2:2020 [20]. Early decay time (EDT_0.5–1kHz_) also is in the optimal range as suggested by Bradley [44]. Clarity value (C50_0.5–2kHz_) satisfies the criteria specified in the UNI 11532-2:2020 [20] standard for classrooms that have a volume of less than 250 m^3^, and definition value is in the optimal range suggested by Marshall [45]. The acoustical treatment resulted in an improvement in all parameter values, with a decrease in reverberation time and an increase in speech clarity and definition. In contrast, the measured data in the AO condition failed to meet any of the standards mentioned above.

Table 1 presents additional information on the acoustics of the two classrooms, including the critical radius (r_c_) and the corresponding five-time distance. The critical radius of each room was determined using the formula r_c_ = √(0.0032 V/T), where V represents the room volume and T represents the reverberation time. This value signifies the sound-source distance at which the intensity of both the direct and reverberant fields are equal. Houtgast et al. [51] proposed that when the distance from the source exceeds five times the critical radius, which can be calculated as 0.3√(V/T), speech intelligibility is influenced solely by the reverberant field.

The distance between the target and receiver as well as the noise and receiver in both classrooms exceed the critical radius. In the AO condition, the critical radius is exceeded five times for two receiver positions and for two noise-to-receiver distances. This highlights that the reverberant field in the AO condition is predominant.

### 3.2. Speech Intelligibility Results 

#### 3.2.1. Effect of Classroom Acoustics

**Figure 2:**
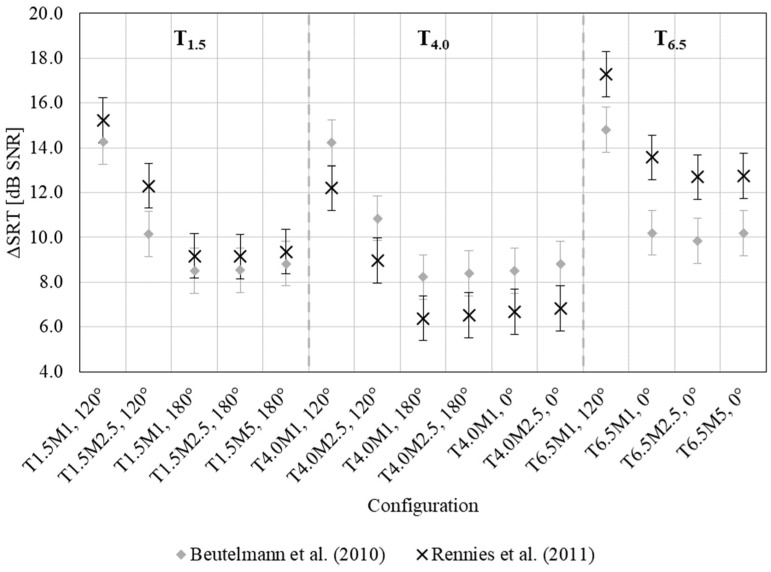
The differences in speech reception thresholds (ΔSRT) between the AO and PO conditions are examined based on the target-to-receiver positions (T1.5, T4.0, T6.5) and the masker-to-receiver distance (N1, N2.5, N5) and angle (0°, 120°, 180°). The differences are shown for both versions of BSIM of Beutelmann et al. [29] and of Rennies et al. [28]. The error bars correspond to just-noticeable difference for sound intensity, adopted from the literature [43].

From Figure 2, it can be observed that intelligibility benefits from the acoustical treatment in all the considered target–masker configurations, distinguishing between the BSIM versions, i.e., Beutelmann et al. [29] and Rennies et al. [28]. Speech intelligibility improved in PO in all scenarios, as evidenced by predicted SRT improvements (ΔSRT, which is calculated as the arithmetic differences between SRT values in the AO and PO conditions). The error bars in Figure 2 are adopted from the literature [43] and correspond to the just-noticeable level differences (JND) measured with normal-hearing listeners. They serve as an orientation for interpretation of the simulated SRTs, i.e., changes or differences in SRT smaller than JND can be treated as not significant; on the contrary, it can be assumed that SRT changes larger than JND are perceptually relevant. BSIM simulations show that the SRT’s improvement after acoustical treatment ranges from 6.4 dB SNR (in T_4_._0_M_1, 180°_ condition) to 17.3 dB SNR (in T_6_._5_M_1, 120°_ condition). Particularly, the BSIM predictions, with their intrinsic variability (i.e., related to the JND applicable to the SRT quantity), allow for understanding whether a specific listening scenario is more or less challenging, as smaller or relevant changes in SRT values are detected with the simulations themselves. For all target-to-receiver distances, the spatially separated noise position yields larger benefits, ranging from 9 to 17 dB SNR improvement. As per [28], there is a more significant enhancement in SRT when the masker is positioned closer to the receiver. Here, at 1 m distance, the improvement is 15.2 dB SNR, while at 2.5 m, it is 12.3 dB SNR. SRT benefits are also observed when the noise source is co-located at 0° and 180° with respect to the receiver. In these cases, there is no influence of the distance of the masker, meaning that the advantage in SRT remains constant regardless of an increase in masker distance. 

Comparing the results of SRTs from [28,29], some discrepancies emerge. While the ΔSRT obtained for the left-hand configuration in Figure 1 with the receiver positions at 1.5 m away from the target source are almost equivalent, i.e., they are within the JND that corresponds to 1 dB for sound intensity [28], the values for the central and right-hand configurations in Figure 1, i.e., when the receiver is at 4.0 m and 6.5 m away from the target, respectively, differ. In particular, in [28], the mean benefit obtained after the acoustical intervention is around 2 dB SNR lower for the central configuration and around 3 dB higher for the right-hand scheme. These differences originate from the predictions that the two models provide for the AO condition, since almost-equal values are given for the PO. In the AO condition, SRTs are predicted to be worse (i.e., higher) in [28], especially for the right-hand configuration, where the target-to-receiver distance is the largest, so that a larger benefit is obtained after the acoustical intervention. This is due to more energy in the late part (>100 ms) of the BRIR of the target speech signal in the right-hand configuration. This effect can be captured using the model of Rennies et al. [28], which predicts higher (worse) SRTs in this condition than the model described in Beutelmann et al. [29]. Focusing again on the AO condition, but considering the central scheme, SRTs provided by [28] are better (i.e., lower) compared to those in [29]. This can be explained by the fact that a model, which can account for the degrading influence of reverberation on the speech signal with increasing distance, is more likely to predict the benefit that listeners in the central positions of the classroom can have due to the support of the early reflections to the speech signal. Moreover, the BRIR of the target is slightly different for the left and right ear due to the physical and acoustic properties of the classroom. The model provided by Rennies et al. [28] can more effectively use this difference since the target does not contain the late reflections that lead to the decorrelation of the signals across the ears and through that affect the effectiveness of binaural processing.

Overall, the results of this study support the findings of previous research [28,29,30], where it was observed that SRTs increased (got worse) with the reverberation. Considerable speech intelligibility improvements were also noted when the reverberation time was short for the monaural acoustic measures, as shown in Table 1. The results of this study demonstrated higher D50 values in all frequency bands in the PO condition (125 Hz: 0.58, 250 Hz: 0.80, 500 Hz: 0.87, 1000 Hz: 95, 2000 Hz: 95, 4000 Hz: 96, 8000 Hz: 97) compared to the AO condition (125 Hz: 0.33, 250 Hz: 0.44, 500 Hz: 0.44, 1000 Hz: 44, 2000 Hz: 59, 4000 Hz: 67, 8000 Hz: 87). These findings suggest that the D50 values are better indicators of intelligibility in the PO conditions, in agreement with [26,29]. Last, considering the SRT results per se, they corroborate the findings from other studies. Westermann and Buchholz [36] proved that the mutual positioning of target and noise source have a significant role in the listening experience when it happens in complex acoustic environments. Particularly, this might bring a large variability across subjects: although this aspect cannot be directly compared to the findings of this study as they relate to model predictions, such an outcome underlines the competitive effect that reverberation and noise bring to the estimation of speech intelligibility under real listening conditions.

#### 3.2.2. Spatial Release from Masking at an Angle

**Figure 3:**
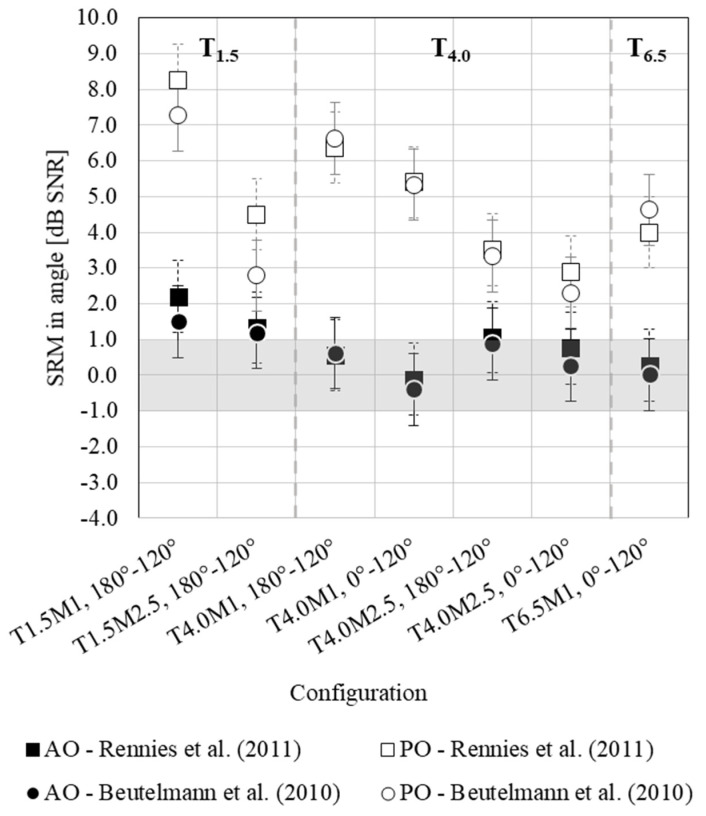
Spatial release from masking (SRM) of close target and masker (T_1.5_M_1,180°–120°_), close target and far masker (T_1.5_M_2.5, 180°–120°_), far target and close masker (T_4.0_M_1, 180°–120°_; T_4.0_M_1, 0°–120°_), far target and far masker (T_4.0_M_2.5, 180°–120°_; T_4.0_M_2.5, 0°–120°_), and very far target and close masker (T_6.5_M_1, 0°–120°_). Filled black symbols indicate the AO condition while the unfilled ones are for the PO condition for both BSIM versions (of Beutelmann et al. [29] and of Rennies et al. [28], indicated with circles and squares, respectively). Error bars indicate the just-noticeable difference for sound intensity, adopted from the literature [43].

Figure 3 indicates a higher benefit of SRM at an angle in the PO than in the AO condition, with a range of the benefit from 2.3 to 8.3 dB SNR, with the greatest benefit in T_1_._5_M_1,180°–120°,_ versus a range between −0.4 and 2.2 dB SNR, respectively. In both PO and AO conditions, moving the masker from 180° to 120° provides greater benefits compared to moving it from 0° to 120°.

The findings imply that the presence of reverberation significantly diminishes the efficacy of binaural cues, which aid in enhancing speech intelligibility in spatially separated conditions. Reverberation causes the signals at the left and right ear to decorrelate, preventing the human binaural system from exploiting binaural cues. As a result, the SRTs in co-located and spatially separated settings are comparable, resulting in either no or significantly reduced SRM. When examining only the setups where the masker is situated 1 m away from the receiver, the SRM measures 6 dB in the PO condition, while in the AO condition, it is notably lower, ranging from 0.4–0.7 dB. This reflects the importance of reverberation for the auditory perception of sound localization in three-dimensional space and quantifies the potential benefits of acoustical intervention in terms of SRM. These findings have significant implications for the acoustical design and treatment of classrooms, highlighting the crucial role of the binaural aspects in source segregation, enabling the focus on the speech target while simultaneously suppressing interfering sources. However, achieving this is only possible in classrooms with good acoustics. This study is limited to the comparison of acoustically untreated and treated classrooms. Future investigations could extend these findings and consider evaluation of the model for different designs of acoustical treatment including different positioning of acoustic surfaces and their acoustic properties. Model simulations should be validated by empirical speech intelligibility measurements to confirm the model outcomes.

As far as the comparison between the results of SRMs from [28,29] is concerned, no difference emerges except for the condition T_1_._5_M_2.5,180°–120°_ in PO that presents higher (better) values in the case of [28]. One possible explanation for this, which is a phenomenon that still needs to be explored further, might be related to the effect of IR separation. Even though the sound present in the late reflections is theoretically negligible, some energy might be left in the IRs, thus affecting the final SNRs. 

Lastly, when comparing the SRM predicted in this study to the results of previous research, which were conducted under anechoic conditions using a speech source positioned in front of the listener and a noise source at varying azimuths [22], several insights can be drawn. As expected, SRMs in complex acoustic scenarios are lower (worse) than those in anechoic environments. At a 120° noise azimuth, it is possible to achieve up to a 13 dB SRT improvement in an environment with no reverberation [22], while this value is reduced to 6 dB in rooms with acoustics such as the one of the PO condition and almost non-existent in rooms with high reverberation such as in the AO condition. This finding is consistent with the results of other studies that have investigated SRM in the presence of reverberation. For instance, in study [26], which considered an office and a cafeteria, and in study [29], which investigated a classroom, a listening room, and a church, it was found that the SRM at 120° was within the range of 2–8 dB. This suggests that the SRM is considerably lower (worse) in reverberant conditions compared to an anechoic condition. Such an outcome is also consistent with the results of Kidd et al. [52] and of a recent paper by Justine Hui et al. [53]. Particularly, in [52], under energetic masking noise, the maximum SRM that was found consisted of up to 8 dB and 2 dB under less and more reverberant conditions, respectively.

## 4. Conclusions

The objective of this study was to examine how normal-hearing individuals process sound inputs from both ears through predicting their ability to comprehend speech in classroom settings with and without acoustical treatment, in the presence of an energetic masking noise. Particularly, speech intelligibility was assessed via the application of a prediction model without involving real listeners. The findings indicated the following:

- Predictions were also in line with D50 values verified in each frequency band.
- The predicted SRTs were lower (indicating better intelligibility) in the classroom that had undergone acoustical treatment, as expected.
- When the noise source was spatially separated from the listener’s head, SRTs were lower (indicating better intelligibility) than when the noise source was spatially co-located. Particularly, considering a noise source position close to the receiver, SRM was found to be up to 8 dB SNR, 6.0 dB SNR, and 4.5 dB SNR for close, far, and very far target-to-receiver distances, respectively.
- SRM improved significantly because reduced reverberation has a direct impact on the listener’s ability to utilize interaural differences between the desired and interfering signals.
- An extended version of the binaural speech intelligibility model (BSIM) which considers the detrimental effect of late reflections on target signal is beneficial for speech intelligibility predictions with a large distance between the source and the target.

Limitations exist in the present study, which should be considered as open questions to be included in future research. The main ones consist of the facts that (i) deeper insights that consider empirical data in the study of complex acoustic scenarios are needed so that the variance across listeners and environmental conditions can be properly evaluated from a robust statistical point of view; (ii) with respect to the above-mentioned limitation, empirical data would also allow for the validation of speech intelligibility thresholds including different listener groups (e.g., adult, child, normal-hearing, hearing-impaired); (iii) BSIM predictions might be used as preliminary tests to establish the most competitive listening scenarios to be tested so that the empirical tests can be kept effective and accurate; (iv) more listening scenarios in terms of reverberation, room dimension, and use should be considered, and further cognitive tasks should be accounted in an overall evaluation so that speech intelligibility predictions can be compared to listening comfort and cognitive performances, too.

In conclusion, ensuring optimal acoustic conditions in classrooms is essential for achieving good speech intelligibility, and the use of accurate prediction tools such as the BSIM can already contribute towards this objective at a design stage. Considering varying classroom typologies at national and international levels, this approach can also be employed for retrofitting existing classrooms through adding sound-absorbing and scattering materials to the walls and ceiling.

## Figures and Tables

**Table 1:** The acoustical parameters measured in AO and PO conditions are summarized using descriptive statistics. Standard deviations are presented in parentheses, and values that meet the standards are emphasized in bold.

Condition	AO	PO	Optimum Value or Range	Reference
T20_0.125–4kHz_ [s]	1.6 (0.1)	**0.6 (0.1)**	~0.5	UNI 11532-2:2020 [20]
EDT_0.5–1kHz_ [s]	1.4 (0.1)	**0.3 (0.1)**	0.3 ÷ 0.7 s	Bradley, 2011 [44]
C50_0.5–2kHz_ [dB]	−0.1 (2.1)	**11.4 (3.7)**	≥2.0	UNI 11532-2:2020 [20]
D50_0.5–1kHz_ [%]	44 (12.8)	**90 (5.0)**	≥86 ÷ 100	Marshall, 1994 [45]
Critical radius (r_c_) [m]	0.7	1.2	n.a.	Houtgast et al., 1980 [51]
$0.3\sqrt{V}/T$ [m]	3.8	6.2	n.a.	

## Data Availability

The data presented in this study are available on request from the corresponding author.
